# Identification of Radil as a Ras binding partner and putative activator

**DOI:** 10.1016/j.jbc.2021.100314

**Published:** 2021-01-20

**Authors:** Byeong Hyeok Choi, Ziyue Kou, Tania Marlyn Colon, Chih-Hong Chen, Yuan Chen, Wei Dai

**Affiliations:** 1 Department of Environmental Medicine, New York University Langone Medical Center, New York, New York, USA; 2 Department of Surgery and Moores Cancer Center, UC San Diego Health, La Jolla, California, USA; 3 Department of Biochemistry and Molecular Pharmacology, New York University Langone Medical Center, New York, New York, USA

**Keywords:** KRas, Radil, cell adhesion, cell invasion, epithelial–mesenchymal transition, DIL, dilute-containing, Dox, doxycycline, EMT, epithelial–mesenchymal transition, ERK, extracellular signal-regulated kinase, FA, focal adhesion, FAK, focal adhesion kinase, FBS, fetal bovine serum, MEK, mitogen-activated protein kinase kinase, RA, Ras-association, RAΔ, RA domain

## Abstract

*Ras* genes are among the most frequently mutated oncogenes in human malignancies. To date, there are no successful anticancer drugs in the clinic that target Ras proteins or their pathways. Therefore, it is imperative to identify and characterize new components that regulate Ras activity or mediate its downstream signaling. To this end, we used a combination of affinity-pulldown and mass spectrometry to search for proteins that are physically associated with KRas. One of the top hits was Radil, a gene product with a Ras-association domain. Radil is known to be a downstream effector of Rap1, inhibiting RhoA signaling to regulate cell adhesion and migration. We demonstrate that Radil interacted with all three isoforms of Ras including HRas, NRas, and KRas, although it exhibited the strongest interaction with KRas. Moreover, Radil interacts with GTP-bound Ras more efficiently, suggesting a possibility that Radil may be involved in Ras activation. Supporting this, ectopic expression of *Radil* led to transient activation of mitogen-activated protein kinase kinase and extracellular signal-regulated kinase; *Radil* knockdown resulted in weakened activation of Ras downstream signaling components, which was coupled with decreased cell proliferation and invasion, and reduced expression of mesenchymal cell markers. Moreover, Radil knockdown greatly reduced the number of adhesion foci and depolymerized actin filaments, molecular processes that facilitate cancer cell migration. Taken together, our present studies strongly suggest that Radil is an important player for regulating Ras signaling, cell adhesion, and the epithelial–mesenchymal transition and may provide new directions for Ras-related anticancer drug development.

Ras proteins comprise a family of small GTPases that are involved in regulating a variety of biological processes including cell survival, proliferation, and migration (1, 2, 3). Ras proteins function as binary signaling switches with “on” and “off” states, which are largely controlled by GTP and GDP binding, respectively (4, 5). Therefore, the activity, subcellular localization, and stability of Ras proteins are tightly regulated in normal cells.

Cell adhesion and motility play a pivotal role in normal development as they are critical for wound healing, stem cell homing, and immune cell trafficking. In normal cells, activated Ras increases cell migration, which is accompanied by extensive actin cytoskeleton remodeling (6, 7, 8). Ras induces changes in interactions between cells and the extracellular matrix (2, 9) and downregulates adhesion junctions including E-cadherin (9). Constitutively activated Ras enhances the motility of transformed cells by altering cytoskeleton structures and deregulating gene expression of adhesion molecules (2, 10).

Epithelial and mesenchymal cells display different features including morphologies, cell-to-cell adhesion, and migratory behaviors (11, 12, 13). During development, cells often switch from a static state to a migratory state or vice versa. For example, wound healing often invokes collective cell migration and epithelial–mesenchymal transition (EMT) in high animals (12, 13). Dysfunctional transition can affect cell growth and differentiation, leading to disease states. At the molecular level, epithelial and mesenchymal cells express distinct sets of gene products, many of which are directly involved in cell adhesion and/or migration (14). E-cadherin, ZO-1, and CK18 are highly expressed in epithelial cells, whereas Snail, Twist, N-cadherin, Zeb1, vimentin, and Claudin-1 are expressed, or highly enriched, in mesenchymal cells (12, 14). EMT is regulated by many proteins including Ras and Y-box–binding protein-1 (14). We have previously shown that expression of KRas^V12^ in MCF7 cells induced expression of Snail and Claudin-1, which is suppressed by the SUMO-resistant counterpart (15), suggesting that Ras sumoylation plays an important role in EMT.

Rap1 is a ubiquitously expressed small GTPase, which plays a key role in modulating cell adhesion, angiogenesis, and endothelial barrier functions (16, 17). Radil is a downstream effector of Rap1, regulating integrin activation and controlling neutrophil chemotaxis (18, 19). Radil consists of three notable domains including Ras-association (RA), dilute-containing (DIL), and PDZ domains (20). DIL domain was originally identified fungi containing a stretch of sequences homologous to the cargo-binding domain of class V myosins. To date, the function of DIL domain remains largely unknown. Upon activation, cytosolic Radil is capable of translocating to the plasma membrane in a Rap1a-GTP–dependent manner (18). Overexpression of Radil causes focal adhesion kinase (FAK) activation and promotes cell adhesion and sustains elongated morphology (18, 21).

In this report, we describe that Radil may function as a new branch in the Ras signaling network. Affinity pulldown coupled with mass spectrometry identified that Radil physically interacted with Ras proteins with KRas exhibiting the highest affinity. The physical interaction between Radil and Ras was influenced by the GTP-bound status of Ras. Radil knockdown led to reduced activation of the mitogen-activated protein kinase kinase (MEK)/extracellular signal-regulated kinase (ERK) signaling axis, which is coupled with decreased cell proliferation, adhesion, and invasion. Moreover, Radil promotes expression of Vimentin, Zeb1, and Snail, transcription factors of the mesenchymal cells.

## Results

### Radil physically interacts with Ras

**Figure 1:**
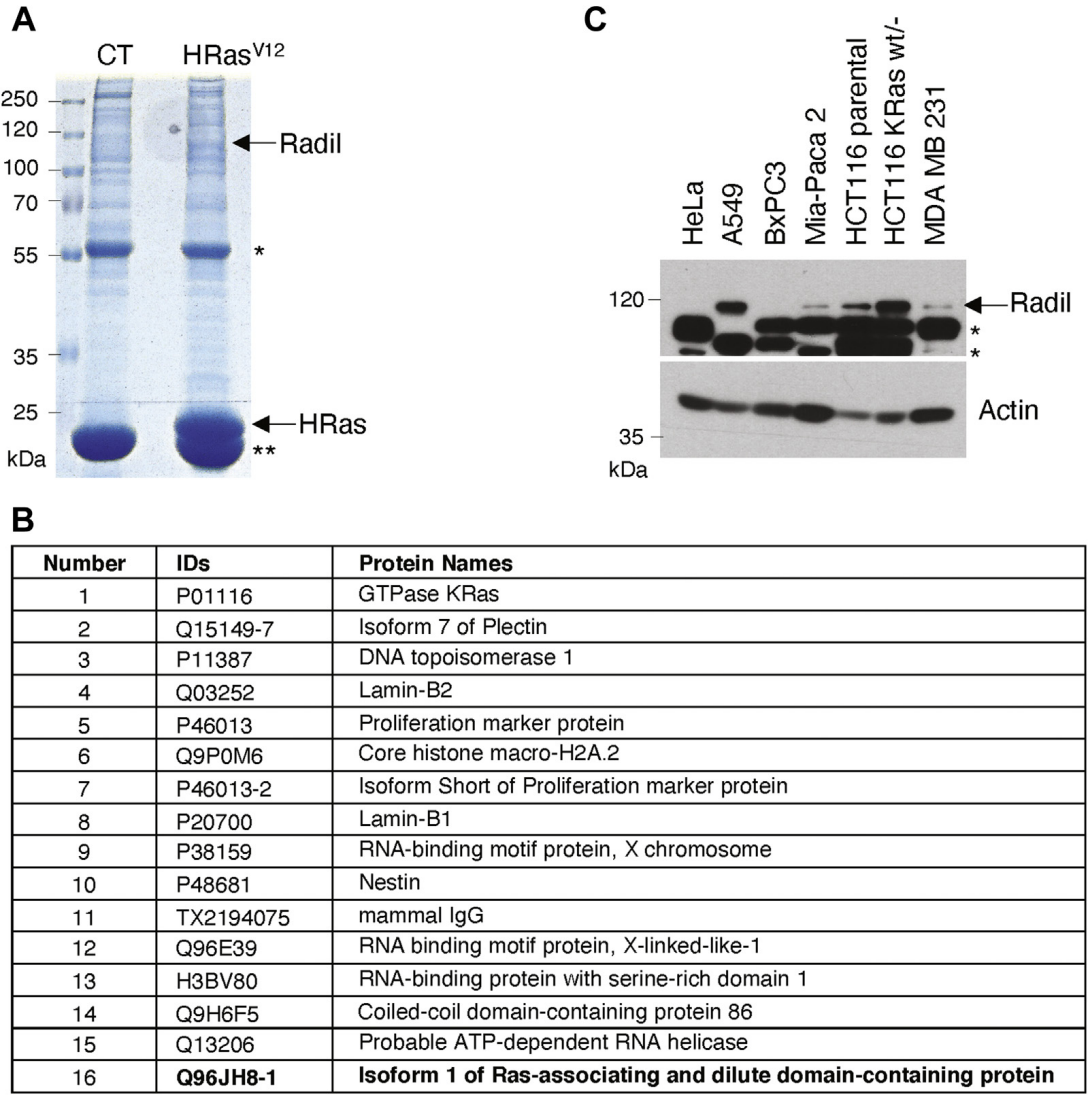
**Radil is a potential Ras-interacting protein.***A*, coomassie Brilliant Blue staining of flag pulldown. HEK293T cells transfected with plasmid expressing HRas^V12^ tagged with Flag or with the empty vector (CT) for 24 h, after which cell lysates were collected for affinity-pulldown using Flag M2 agarose as described in Experimental procedures and Supplemental Procedures. Affinity-purified proteins were subjected to SDS-PAGE analysis. *Arrow* indicates the position of Radil or HRas. The *single star* (∗) indicates IgG heavy-chain band. The *double stars* (∗∗) indicate IgG light-chain band. *B*, affinity-purified proteins were subjected to mass spectrometric analysis. Proteins identified with top scores were listed and Radil was highlighted. *C*, a survey of Radil expression in various tumor cell lines *via* Western blotting. The *arrow* indicates the Radil specific bands. The *star* (∗) denotes nonspecific bands. IgG, immunoglobulin G.

Ras activities are directly mediated by various downstream components it interacts with (22, 23, 24, 25). To further elucidate the function of Ras in regulating cell proliferation and oncogenic transformation, we attempted to identify new molecular components that physically interacted with Ras. We ectopically expressed HRas^V12^ that was tagged with the Flag moiety. Through the use of affinity pulldown and mass spectrometry, we identified a number of candidate proteins that physically interacted with Flag-tagged HRas (Fig. 1, *A* and *B*).

To ascertain which proteins were enriched in the KRas IP over the empty vector control, we calculated a fold change using the spectral counts. To be able to calculate a ratio for proteins that had only been identified in the sample and not the control, we imputed the data by adding 2 spectral counts to all proteins in both samples.

As expected, HRas (the bait) was the highest scored proteins (Table S1). KRas was identified as the top-interacting protein (Fig. 1*B*). Among others, Radil is one of the top-interacting proteins (Fig. 1*B*, highlighted). We first focused on characterizing Radil as it contains interesting domains. Western blotting revealed that Radil expression varied among transformed cell lines with A549 (lung carcinoma) and HCT116 (colorectal carcinoma) exhibiting high levels of expression (Fig. 1*C*).

**Figure 2:**
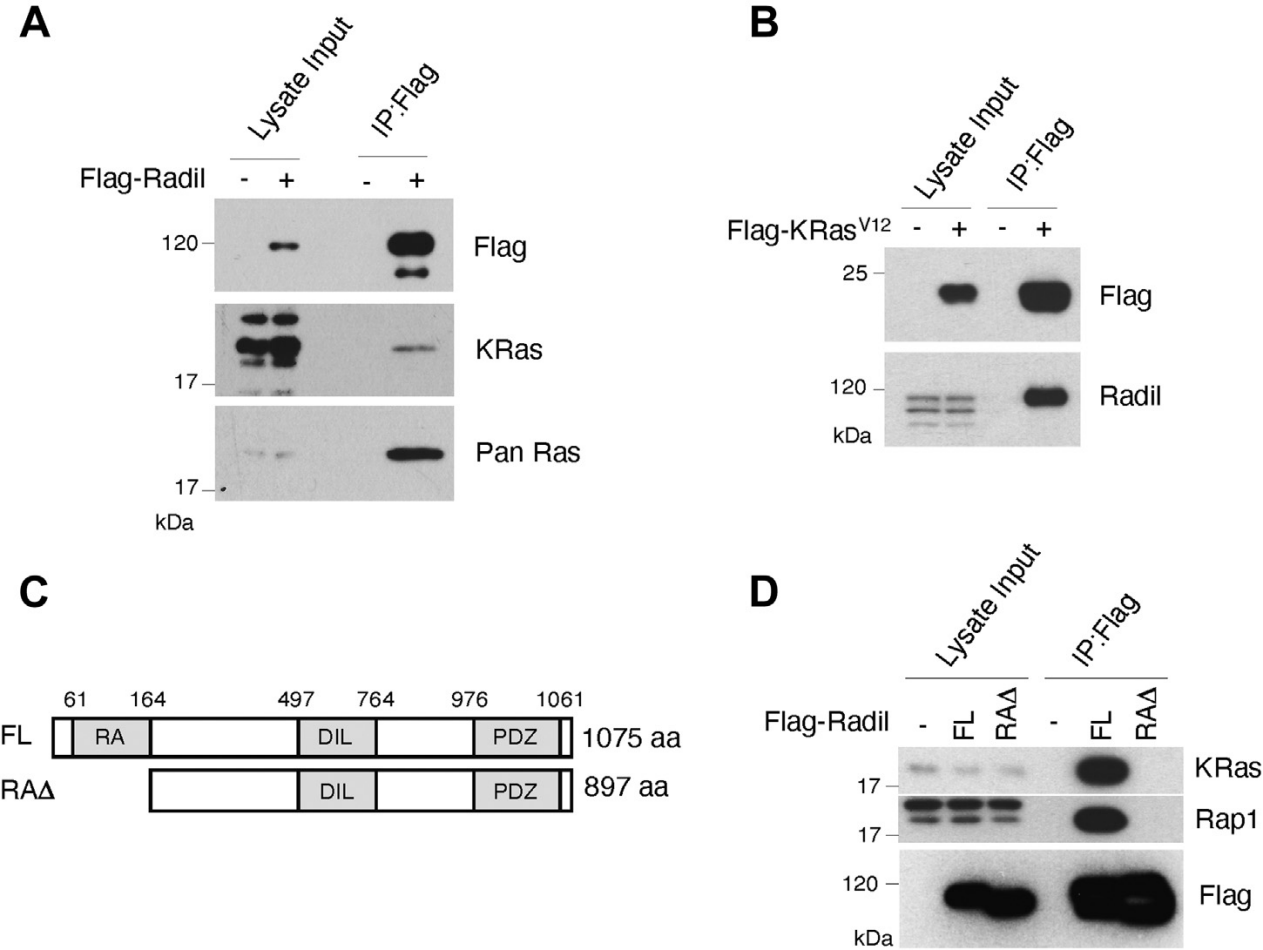
**Radil physically interacts with KRas.***A*, HEK293T cells transfected with Flag-Radil were lysed, and cell lysates were immunoprecipitated with the anti-Flag antibody or control IgG. Flag immunoprecipitates, along with lysate inputs, were blotted with antibodies for Flag, KRas 4b, and Pan-Ras proteins. *B*, HEK293T cells were transfected with the plasmid expressing Flag-KRas^V12^ for 24 h, after which cell lysates were immunoprecipitated with the anti-Flag antibody. Flag immunoprecipitates, along with lysate inputs, were blotted for Radil and Flag. *C*, domain structures of full-length Radil (FL) and its mutant without RA domain (RAΔ). *D*, HEK293T cells transfected with FLAG-Radil FL and RAΔ expression plasmids for 24 h, after which cell lysates were immunoprecipitated with the anti-Flag antibody. Flag immunoprecipitates, along with lysate inputs, were blotted for KRas4B, Rap1, and Flag. IgG, immunoglobulin G.

Given that direct physical association between Ras and Radil has not been reported in the literature, we performed a series of experiments to confirm whether Radil is a *bona fide* Ras-interacting protein. We transfected HEK293T cells with Flag-Radil plasmid for 24 h, after which cells were collected and lysed for coimmunoprecipitation (Co-IP) analysis using the anti-Flag antibody. Flag-Radil was efficiently expressed and recovered by immunoprecipitation (Fig. 2*A*). Endogenous Ras proteins were enriched in the immunoprecipitates after blotting with a pan-Ras antibody. Endogenous KRas4B (KRas thereafter) was also pulled down with Flag-Radil, suggesting that Radil physically associates with KRas. As a reciprocal approach, we transfected HEK293T cells with a Flag-KRas^V12^ expression plasmid for 24 h, after which the cell lysates were immunoprecipitated with the Flag antibody. Co-IP analysis revealed that a significant amount of Radil was enriched in Flag-KRas^V12^ precipitates (Fig. 2*B*). These combined results thus indicate that Radil physically interacts with KRas.

Radil protein consists of RA, DIL, and PDZ domains (Fig. 2*C*). The RA domain (RAΔ) has been found in several other Ras-interacting proteins including Rin1, RASSF5, RalGDS, PLCs, and Ras GEFs (26). In fact, it is essential for mediating the interaction between Radil and Rap1, the latter being a small G-protein that plays an important role in the regulation of endothelial function (17). We then determined whether it was also required for mediating its interaction with Ras. We observed that whereas the full-length Radil was capable of pulling down both KRas and Rap1 (Fig. 2*D*), the deletion of the RAΔ completely abolished the interaction between Radil and Ras (and Rap1). Expression of Flag-Radil and Flag-Radil^RAΔ^ was equally efficient (Fig. 2*D*). These results indicate that Radil physically interacts with Ras and that RAΔ of Radil is essential for mediating the interaction.

### Radil binds more efficiently to GTP-bound Ras

**Figure 3:**
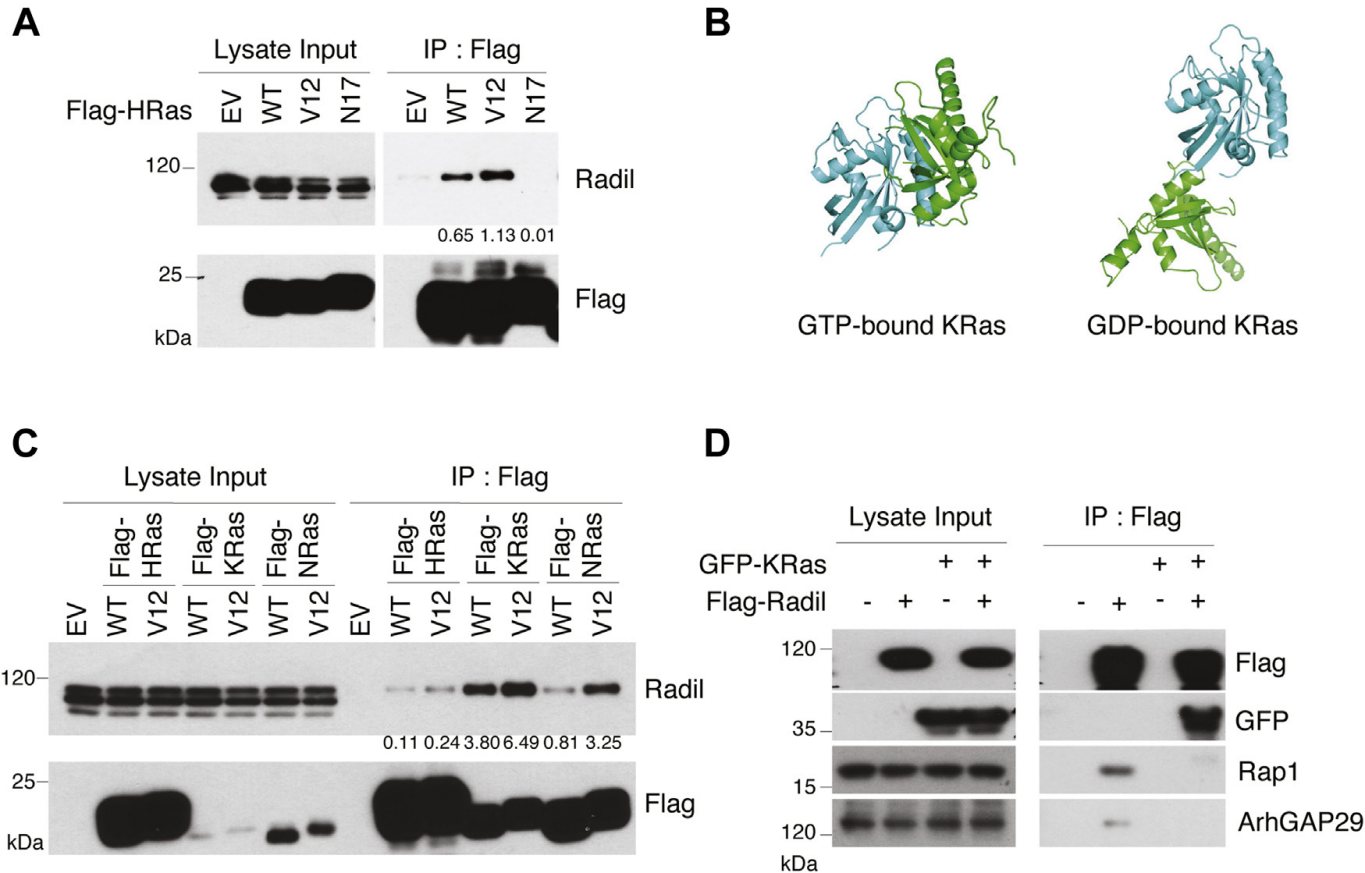
**Radil interacts with active Ras proteins.***A*, HEK293T cells were transfected with the plasmid expressing Flag-tagged HRas (WT), constitutively active HRas (V12), or dominant-negative HRas (N17) for 24 h, after which cell lysates were immunoprecipitated with the anti-Flag antibody. Flag immunoprecipitates, along with lysate inputs, were blotted for Radil and FLAG. The amount of Radil interacting with various forms of KRas was quantified *via* densitometry. Normalized values equal the density of Ras-bound Radil/density of total Radil/fold density of IP Flag. *B*, computer modeling structures of KRas/Radil interaction with or without GTP binding. *C*, HEK293T cells were transfected with plasmids expressing either WT or constitutively active forms of Ras (V12) proteins as indicated for 24 h, after which cell lysates were immunoprecipitated with the anti-Flag antibody. Flag immunoprecipitates, along with lysate inputs, were blotted for Radil and Flag. The amount of Radil interacting with various forms of KRas was quantified *via* densitometry. Normalized values equal the density of Ras-bound Radil/density of total Radil/Fold density of IP Flag. *D*, HEK293T cells were transfected with plasmids expressing GFP-KRas and/or Flag-Radil for 24 h, after which cell lysates were immunoprecipitated with the anti-Flag antibody. Flag immunoprecipitates, along with lysate inputs, were blotted for Flag, GFP, Rap1, and ArhGAP29.

Biochemically, Ras GTPases catalyze the hydrolysis of GTP to GDP. GTP-bound Ras is active, whereas GDP-bound one is inactive (1, 2, 3). To test whether GTP/GDP-loading status affected the interaction between Radil and Ras, we transfected HEK293T cells for 24 h with a plasmid encoding Flag-HRas (WT HRas), constitutively active mutant (HRas^V12^ bound with GTP), or dominant negative mutant (HRas^N17^ incapable of GTP-binding). Co-IP with the Flag antibody followed by immunoblotting revealed that more Radil was associated with HRas^V12^ than with HRas (Fig. 3*A*), strongly suggesting that GTP binding affects the Radil–Ras interaction. Further supporting this notion, no Radil was detected in HRas^N17^ immumoprecipitates (Fig. 3*A*). Expression of transfected plasmids coding for various Ras proteins was comparable, and the immunoprecipitation was efficient as well. Therefore, these observations strongly suggest that Radil may positively regulate or mediate Ras activation and signaling. Ras binds to GTP, or GDP results in conformational changes (4). We speculated that Radil may prefer a GTP-bound Ras conformation. To test this hypothesis, we simulated 3D conformation of Radil-RA–KRasGTP and Radil-RA–KRasGDP complexes using available software. We found that Radil displays a higher affinity to GTP-bound KRas than to GDP-bound one (Fig. 3*B*).

### Radil preferentially interacts with KRas

Downstream effectors that mediate Ras activities are frequently isoform specific (27). To investigate whether Radil binds to different isoforms of Ras with a different affinity, we transfected HEK293T cells with a plasmid construct coding for Flag-tagged HRas, NRas, or KRas. Plasmids expressing constitutively active mutants (V12) of each isoform were also used as controls. We observed that Radil interacted with WT KRas more efficiently than with either WT NRas or WT HRas although KRas expression was very low compared with that of either NRas or HRas (Fig. 3*C*). Consistent with above observations, an enhanced interaction was detected between Radil and constitutively active Ras (HRas^V12^, KRas^V12^, and NRas^V12^), given that expression of WT Ras and active Ras of various isoforms was comparable (Fig. 3*C*).

We next asked how KRas might mediate the interaction with Radil. Cells transfected with plasmids expressing GFP-KRas and/or Flag-Radil were lysed, and cell lysates were immunoprecipitated with the anti-Flag antibody. We observed that Rap1 and ArhGAP29 were present in Radil immunoprecipitates, indicating that Radil constitutively interacted with Rap1 and ArhGAP29 (Fig. 3*D*). Radil also strongly interacted with GFP–KRas as Radil immunoprecipitates contained a high level of GFP-KRas. Interestingly, the interaction between KRas and Radil suppressed the interaction between Radil and Rap1 or ArhGAP29, suggesting that KRas competes with Rap1 and ArhGAP29 for Radil.

### Radil regulates Ras signaling pathway

**Figure 4:**
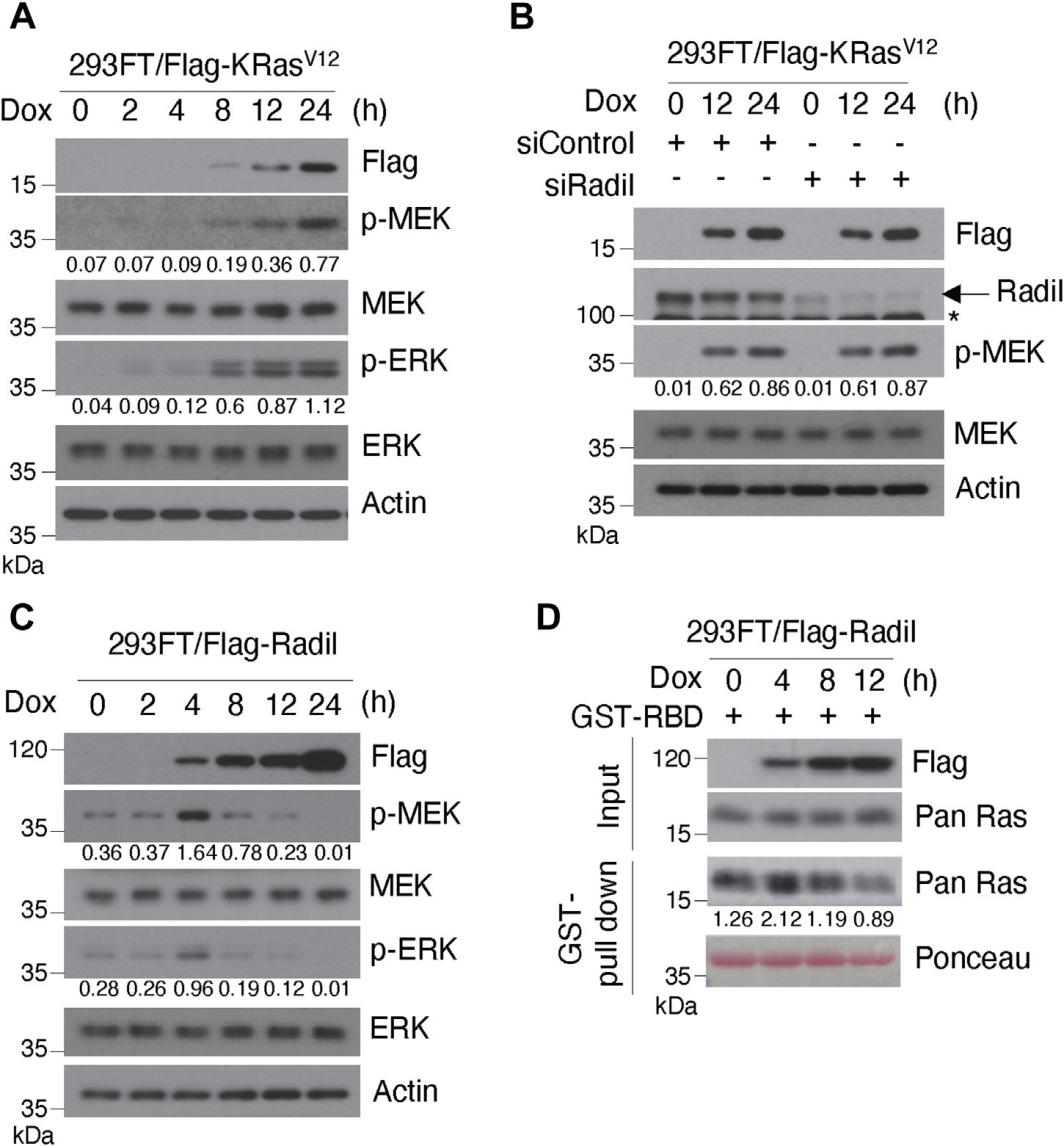
**Radil positively regulates KRas signaling.***A*, 293FT cells engineered to express inducible Flag-KRas^V12^ (293FT/Flag-KRas^V12^) were starved in low-FBS (0.2%) medium for 16 h and then treated with doxycycline (Dox) for various times and cell lysates were blotted for Flag, phospho-MEK, MEK, phospho-ERK, ERK, and actin. Signals of p-MEK and p-ERK were quantified by densitometry. Values presented are from the calculation of density of phospho-MEK (or p-ERK)/density of total MEK (or ERK). *B*, 293FT/KRas^V12^ cells were transfected with Radil-specific siRNA (siRadil) and/or nontargeting control pool siRNAs (siControl) for 12 h and then treated with Dox 12 and 24 h. Cell lysates were then blotted for Flag, Radil, phospho-MEK, MEK, and actin. p-MEK signals were quantified by densitometry. The *arrow* indicates the position of Radil. Values presented are from the calculation of the density of phospho-MEK normalized by the density of total MEK. ∗ indicates nonspecific bands. *C*, 293FT cells engineered to express inducible Flag-Radil (293FT/Flag-Radil) were treated with Dox for various times as indicated and cell lysates were blotted for Flag, phospho-ERK, ERK, phospho-MEK, MEK, and actin. Signals of p-MEK and p-ERK were quantified by densitometry. Values presented are from the calculation of the density of phospho-MEK (or p-ERK) divided by the density of total MEK (or ERK). *D*, 293FT/Flag-Radil cells were treated with Dox for various times as indicated. Equal amounts of lysates were subjected to the *in vitro* pull-down analysis using Raf-RBD, which specifically interacts with GTP-bound Ras (GST-RBD). The amount of GTP-Ras pulled-down was quantified by sensitometry. The proteins subjected to GST pull-down assay were stained with Ponceau S staining. The values presented are derived from the density of active Ras normalized by the density of total Ras (Pan Ras). FBS, fetal bovine serum.

Given that the association between Radil and Ras is affected by the GTP-binding status of Ras, we hypothesized that Radil is involved in regulating Ras activity and signaling. To test this possibility, we generated stable cell line expressing inducible KRas (293FT/Flag-KRas^V12^). Upon doxycycline (Dox) treatment, expression of transfected FLAG-KRas was steadily induced, leading to the activation of downstream signaling components including p-MEK and p-ERK (Fig. 4*A*).

We then asked the role of Radil in KRas^V12^-activated downstream activation. 293FT/Flag-KRas^V12^ cells were transfected with Radil siRNAs or control sRNAs and then treated with Dox for 12 or 24 h, after which cells were analyzed for expression of transfected KRas and endogenous Radil, as well as Ras downstream signaling. We observed that knocking down Radil had no significant effect on the activation/phosphorylation of MEK by ectopically expressed KRas^V12^ (Fig. 4*B* and Fig. S1*D*). To further elucidate the role of Radil in modulating Ras signaling, we generated stable cell line with inducible expression of Flag-Radil (293FT/Flag-Radil). Upon Dox treatment, Flag–Radil expression was rapidly and steadily induced (Fig. 4*C*). Increased expression of Flag–Radil was correlated with transient activation of MEK and ERK, peaking around 4 h after Dox treatment (Fig. 4*C*). Intriguingly, further increase in Radil expression was correlated with suppression of p-MEK and p-ERK.

To elucidate the possible mechanism of Ras regulation by Radil, we measured the level of GTP-bound Ras after induced Radil expression. We observed that treatment with Dox for about 4 h induced Radil expression, which was coupled with increased GTP–Ras as well (Fig. 4*D*). On the other hand, continued induction of Radil deceased the level of GTP–Ras, strongly suggesting that Radil may regulate Ras activity in a concentration-dependent manner.

**Figure 5:**
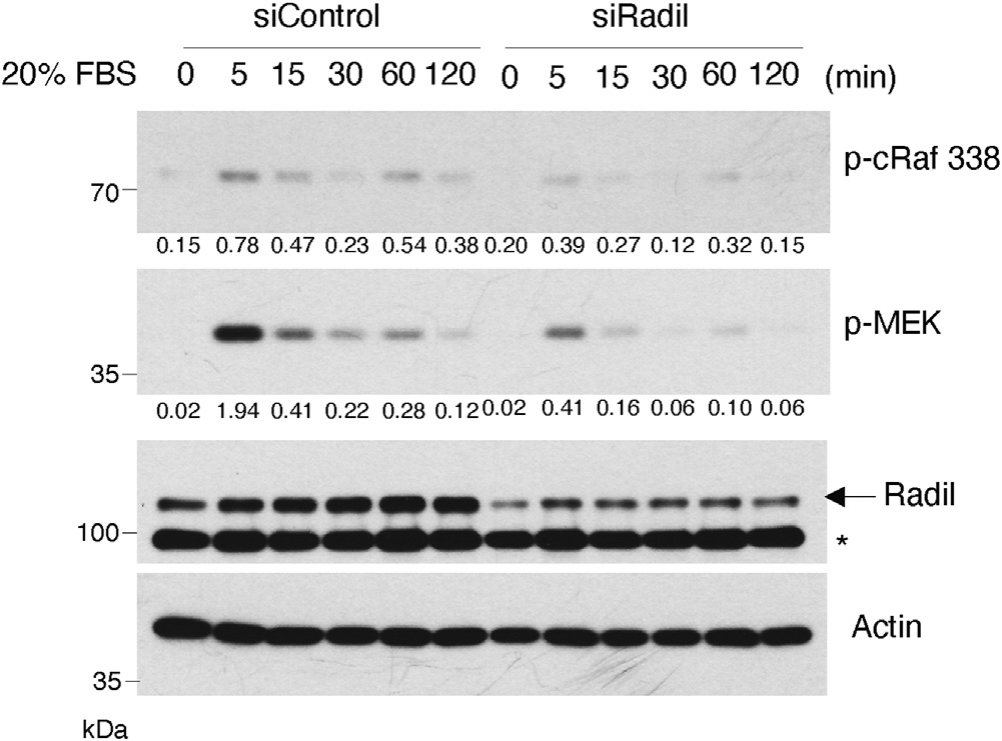
**Radil mediates growth factor–induced activation of cRaf and MEK.** A549 cells were transfected with Radil siRNAs (siRadil) or nontargeting control pool siRNAs (siControl) for 24 h, starved in low (0.2%) FBS medium for 16 h, and then fed 20% serum for various times as indicated. Cell lysates were then blotted for Radil, phospho-cRaf 338, phospho-MEK, and actin. Signals of p-cRaf and p-MEK were quantified. The *arrow* indicates the position of Radil. The values presented are derived from the density of phospho-form normalized by the density of actin. ∗ indicates nonspecific bands. FBS, fetal bovine serum.

We next determined whether knocking down the basal level of Radil affected Ras downstream signaling. Cells transfected with siRNAs to either control or Radil for 24 h after which cells were fed 20% fetal bovine serum (FBS) for various times. We observed that Radil downregulation compromised phosphorylation/activation of cRaf and MEK by growth factors in a time-dependent manner (Fig. 5 and Fig. S1C). These results strongly suggest that Radil is involved in regulating Ras downstream signaling.

### Radil regulates EMT and cell invasion

**Figure 6:**
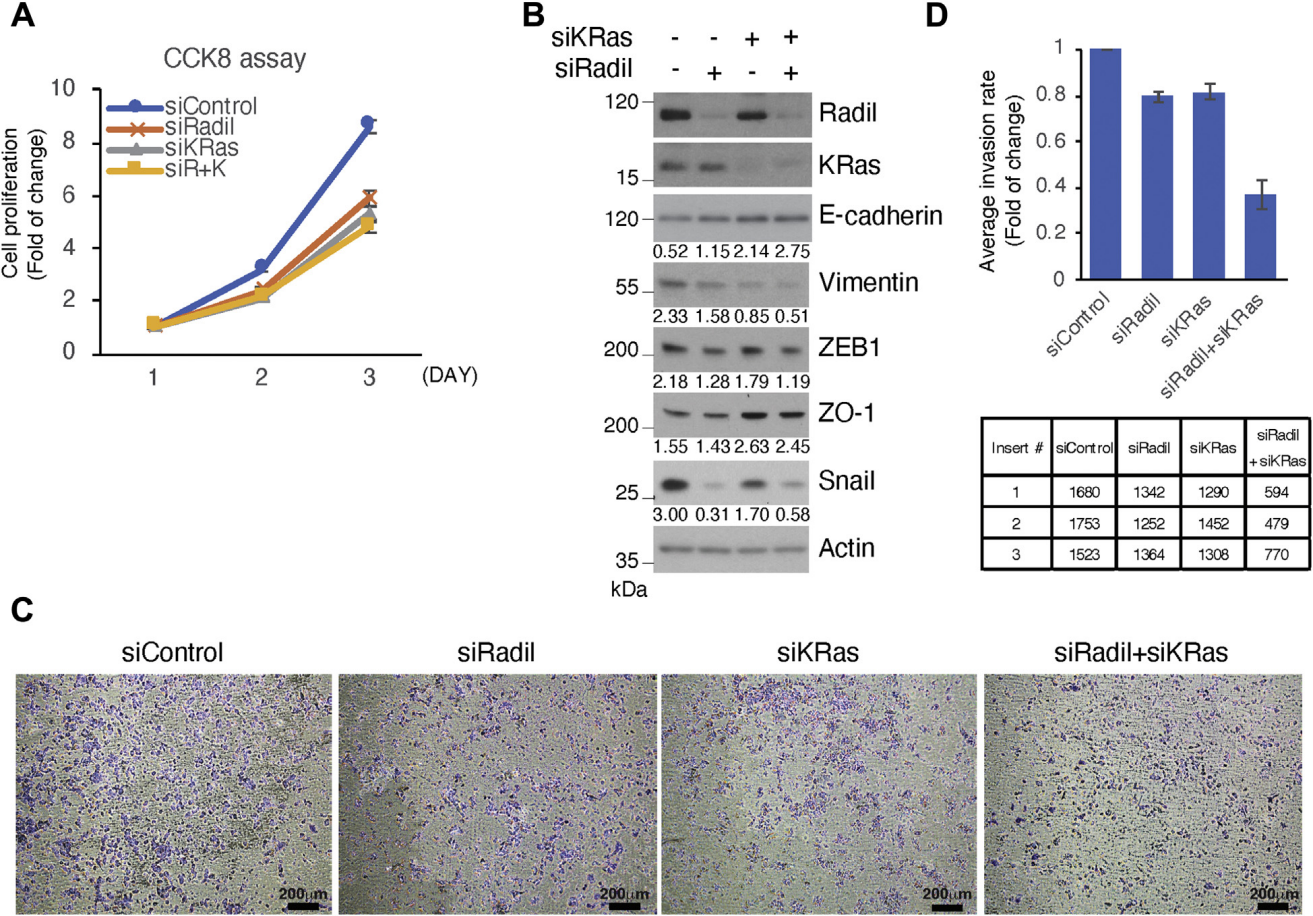
**Radil positively regulates cell proliferation, migration, and EMT.***A*, A549 cells transfected with Radil siRNAs (siRadil), KRas siRNAs (siKRas), and combination of Radil and KRas siRNAs (siR+K) for various times as indicated. Control cells were transfected for the same length of time with nontargeting control pool siRNAs (siControl). Cell proliferation was measured as described in Experimental procedure. *B*, A549 cells transfected with Radil siRNAs and/or KRas siRNAs for 24 h. Cell lysates were then blotted for Radil, KRas, E-cadherin, Vimentin, Zeb1, ZO-1, Snail, and actin. Signals of E-cadherin, vimentin, ZEB1, ZO-1, and Snail were quantified by densitometry. The values presented are derived from the density of each protein normalized by the density of actin. *C*, representative images of transwell invasion assays. A549 cells transfected with Radil siRNAs and/or KRas siRNAs for 24 h were seeded in Matrigel for cell-invasion assay. Cells transfected with nontargeting control pool siRNAs (siControl) were used as control. Scale bar = 200 μm. *D*, quantification of transwell invasion assays (*top panel*) and total counts of invaded cells in three individual images from transwell inserts (*bottom panel*). Data on cell invasion after various treatments were summarized and quantified. Data represent the mean (±standard deviation, SD) of three independent experiments, each performed in triplicate and are presented relative to control. Error bars indicate SDs. *Stars* indicate statistical significance at *p* < 0.05, ∗; and *p* < 0.01, ∗∗. EMT, epithelial–mesenchymal transition.

We have previously shown that KRas plays an important role in regulating cell migration, invasion, and EMT (15). To determine whether Radil might be also involved in these processes, we knocked down Radil and/or KRas in A549 cells and then measured their proliferation rate. We observed that compared with the control cells, knocking down either Radil or KRas significantly reduced cell proliferation (Fig. 6*A*). Immunoblotting revealed that Radil knockdown greatly reduced expression of vimentin (Fig. 6*B* and Fig. S1B), a mesenchymal marker positively associated with motility and adhesion (28, 29, 30). Radil knockdown significantly reduced the expression of Snail and Zeb1, two master transcription factors for mesenchymal cells, with a concomitant increase of E-cadherin, an epithelial cell marker (Fig. 6*B*). As expected, knockdown of KRas also caused downregulation of vimentin and snail, which was correlated with upregulation of ZO-1, an epithelial cell marker (Fig. 6*B*). Further supporting EMT induced by Radil and KRas, we carried out cell invasion assays after downregulation of Radil and KRas. We observed that knockdown of Radil or KRas led to reduced cell invasion and that the effect of decreased cell invasion by downregulation of Radil and Ras was additive (Fig. 6, *C* and *D*).

### Radil and KRas regulate formation of focal adhesions and actin polymerization

**Figure 7:**
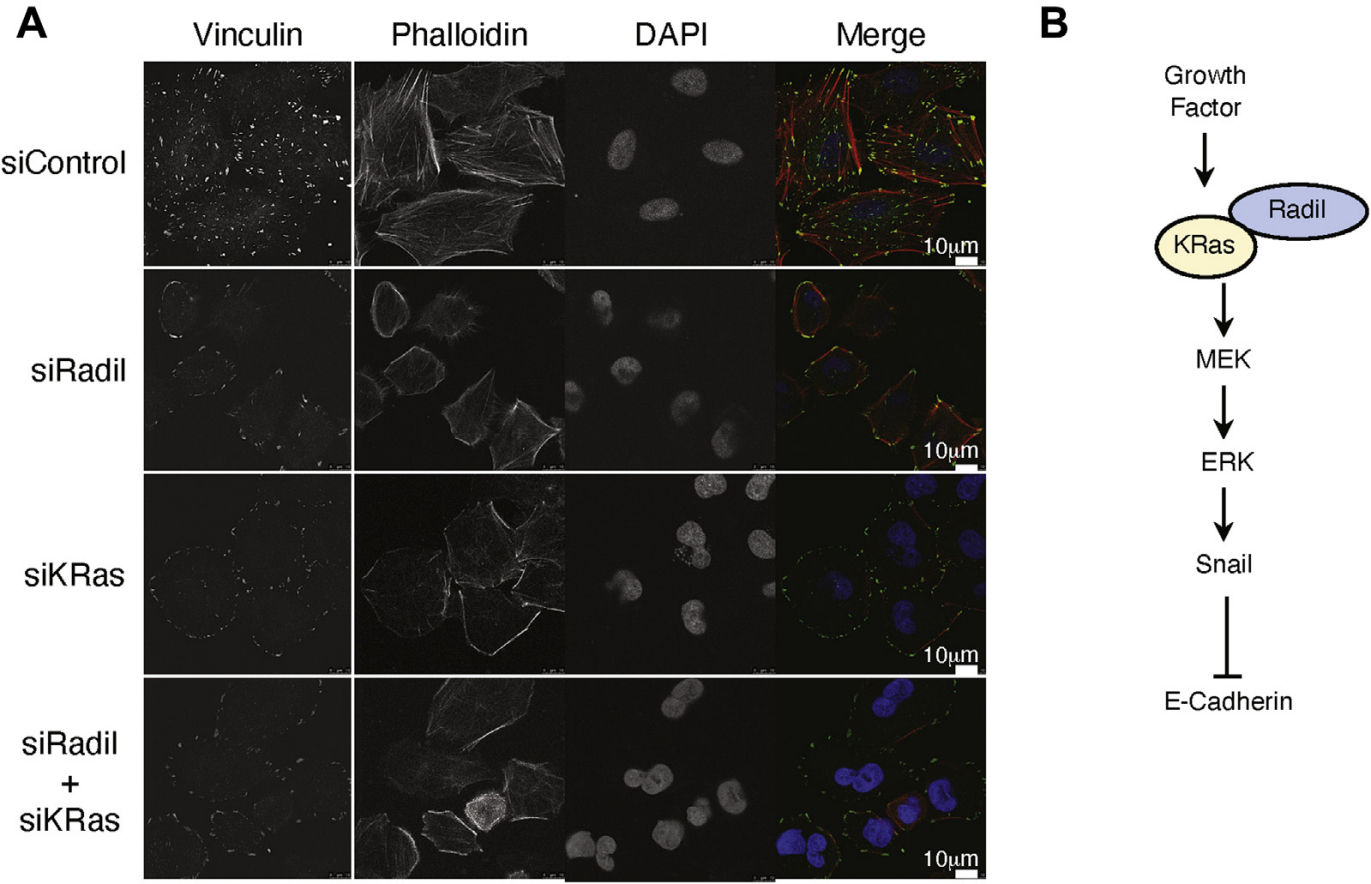
**Radil regulates cell adhesion and actin dynamics.***A*, A549 cells seeded on chamber slides were transfected with Radil siRNAs (siRadil) and/or KRas siRNAs (siKRas) for 24 h. Control cells were transfected for the same length of time with nontargeting control pool siRNAs (siControl). Cells of various treatments were fixed and stained with the anti-vinculin antibody and phalloidin. Representative images are shown. *B*, a model that depicts Radil’s function in regulating KRas signaling in response to growth factors during EMT. EMT, epithelial–mesenchymal transition.

To further understand how Radil or KRas affects cell proliferation and invasion, as well as EMT, we measured cell adhesion and cytoskeleton organization after modulating either Radil or KRas expression. Cells transfected siRNAs to Radil, KRas, or both were fixed and stained with antibodies to vinculin (a component of focal adhesion [FA]) and phalloidin (staining for F-actin). Compared with control cells, Radil knockdown greatly reduced the number of FAs in the cells (Fig. 7*A*), which was associated with the loss of actin polymerization and a significantly reduced cell size. KRas knockdown also reduced the number of intracellular FAs and actin polymerization; it also affected the cell morphology although the cell size did not seem to be reduced as dramatically as it did with Radil siRNA (Fig. 7*A*). Kinase profiling revealed that expression of KRas^V12^ also activated FAK (Fig. S2), a protein kinase positively involved in integrin-mediated cell adhesion.

Based on the present study, we propose the following model that depicts Radil’s role in modulating KRas during cell proliferation, migration, and EMT (Fig. 7*B*). As a molecular component that mediates the function of growth factors, Radil interacts with and modulates KRas activity. Prolonged activation of KRas and its downstream signaling leads to enhanced expression of mesenchymal cell phenotypes coupled with suppression of epithelial cell phenotypes.

## Discussion

We have identified Radil as a new Ras-interacting protein that plays an important role in regulating KRas activity in cell proliferation, migration, and EMT. We demonstrate that Radil interacts with all three isoforms of Ras including HRas, NRas, and KRas although it exhibits the strongest interaction with KRas. Moreover, Radil interacts with GTP-bound Ras more efficiently than it does with GDP-bound one, suggesting a possibility that Radil may be involved in regulating Ras activity. Supporting this, we show that induced expression of Radil leads to activation of MEK and ERK albeit transiently. In addition, knockdown of Radil compromises activation of Ras downstream components including cRaf and MEK1 by FBS. Intriguingly, high levels of Radil lead to reduced downstream signaling (Fig. 4*C*). This could be due to feedback control of KRas activity by a downstream component(s). Alternatively, high levels of intracellular Radil could promote its interaction with and activation of Rap1, also a small GTPase. Competition between Rap1 and Ras proteins for intracellular GTP would diminish the activation of KRas signaling. Supporting this, we observe that continued induction of Radil reduces the amount of GTP-bound Ras (Fig. 4*D*).

Radil-mediated activation of Ras signaling is likely associated with its ability in promoting cell adhesion, migration, and EMT. (i) Radil knockdown decreases cell proliferation and cell invasion; (ii) Radil knockdown compromises expression of mesenchymal cell regulators including Vimentin, Zeb1, and Snail coupled with an increase in expression of epithelial cell marker E-cadherin; (iii) Radil knockdown greatly reduces the number of adhesion foci and actin polymerization, which is correlated with a decreased cell size. EMT is frequently associated with aggressive tumor phenotypes such as invasive and metastatic characteristics (31, 32). Thus, it is likely that aberrant expression and/or mutations of Radil may play an important role in tumor development and progression.

Our studies suggest that Radil displays both KRas-dependent and KRas-independent functions. (i) Radil knockdown plays a more significant role than KRas in suppressing expression of major mesenchymal genes including Zeb1 and Snail (Fig. 6*B*), suggesting that Radil is a major factor that drives cells toward the mesenchymal phenotype. Consistent with reports that Snail suppresses expression of E-cadherin (11, 33), we observe that silencing Radil and/or KRas leads to upregulation of E-cadherin (Fig. 6*B*). (ii) Radil knockdown also greatly shrinks the cell size with disorganized actin filaments as compared with cells with KRas knockdown (Fig. 7*A*). Formation of focal adhesins is essential for cell–cell and cell–matrix adhesins. It is tempting to speculate that Radil is a critical factor that mediates the formation of focal adhesin through physical and functional interactions with major cellular regulators including both KRas and Rap1.

Radil is originally identified as an effector of Rap1, which is a small GTPase regulating cell–cell adhesion, cell–matrix adhesion, and actin structures. Mechanistically, Ras and Rap1are capable of cooperating in initiating and sustaining MEK/ERK signaling, thus positively regulating oncogenesis, especially in promoting invasion and metastasis (17). In this report, we show that Radil interacts with Rap1 through the RAΔ. Given the RAΔ is also crucial for the interaction between Ras and Radil, we have proposed that there is a competition between KRas and Rap1 in interacting with Radil. Supporting it, we demonstrate that enhanced KRas expression compromises the interaction between Rap1 and Radil. High intracellular levels of Radil may favor the interaction between Radil and Rap1, thus diminishing Ras downstream signaling. At present, it remains unclear how KRas and Rap1 coordinate, or compete for, the regulation of cell adhesion and migration. Given that FAK is upregulated by KRas^V12^ (Fig. S1), it is reasonable to speculate that Radil is involved in integrin-mediated cell adhesion and migration through perturbation of the balance between Radil–KRas and Radil–Rap1 complexes.

## Experimental procedures

### Materials

The cell transfection agent, Lipofectamine 3000, was purchased from Thermo Fisher Scientific. DharmaFECT I for siRNA transfection was obtained from Dharmacon. BD Matrigel was purchased from BD Biosciences. Dox, hygromycin, protease inhibitor cocktail, and Flag M2 agarose were obtained from Sigma-Aldrich. Antibodies against Flag, Pan Ras, Rap1, phospho-cRaf 338, phospho-MEK, MEK, phospho-ERK42/44, ERK2, E-Cadherin, Vimentin, ZEB1, ZO-1, Snail, and Actin were purchased from Cell Signaling Technology. Polyclonal Radil and ArhGap29 antibody were purchased from ProteinTech. Polyclonal anti-KRas antibodies were obtained from Santa Cruz Biotechnology. Dulbecco's modified Eagle's medium (DMEM), RPMI 1640, McCoy’s 5A, the FBS, L-glutamine, penicillin, and streptomycin were purchased from Thermo Fisher Scientific. Tetracycline-free FBS was obtained from Atlanta Biological.

### Cell culture

HEK293T (kidney carcinoma), A549 (lung carcinoma), HeLa (cervical adenocarcinoma), Mia-Paca 2 (pancreas ductal adenocarcinoma), and MDA-MB-231 (mammary carcinoma) cell lines obtained from the American Type Culture Collection were cultured in the DMEM supplemented with 10% FBS and antibiotics (100 μg/ml of penicillin and 50 μg/ml of streptomycin sulfate) at 37 °C under 5% CO_2_. BxPC3 cell line obtained from the American Type Culture Collection was cultured in RPMI supplemented with 10% FBS and antibiotics (100 μg/ml of penicillin and 50 μg/ml of streptomycin sulfate) at 37 °C under 5% CO_2_. HCT116 parental and KRas wt/- cell lines obtained from Johns Hopkins University were cultured in McCOY’s 5A medium supplemented with 10% FBS and antibiotics (100 μg/ml of penicillin and 50 μg/ml of streptomycin sulfate) at 37 °C under 5% CO_2_. 293FT (HEK-293 Flp-In T-Rex; Thermo Fisher Scientific) cells were cultured in DMEM supplemented with penicillin–streptomycin and 10% tetracycline-free FBS at 37 °C, 5% CO_2_, and 90% humidity.

### Plasmids

pcDNA/FRT/TO/Flag-Ras plasmids including WT, constitutive-active, or dominant-negative HRas, NRas, KRas4B (KRas thereafter), and GFP-KRas were kindly provided by Dr Mark Philips (New York University Langone Health). Full-length (pcDNA/FRT/TO/Flag-Radil) and RAΔ deletion constructs (pcDNA/FRT/TO/Flag-Radil RA del.) were prepared by standard molecular biology techniques and PCR amplification of the described fragments. Briefly, each construct generated PCR products encoding human Radil FL, or RA deletion was inserted into the Bam HI and EcoRI site of pcDNA5/FRT/TO vector (Thermo Fisher Scientific).

### Establishment of stable cell lines

For stable transfections, 293FT cells were seeded at a density of 2.8 × 105 cells/well in 6-well plates 24 h before transfection. In each well, 2.5 μg of the pcDNA5/FRT/TO vector with the gene of interest and 0.5 μg of the pOG44 vector expressing the Flp recombinase were transfected with the Lipofectamine 3000. Twenty four hours after transfection, the cells were transferred to 10-cm culture dishes. Clones from each transfection were selected and maintained in the medium containing the hygromycin (100 μg/ml). Expression of stable cell lines was induced with 1 μg/ml Dox for 24 h.

### Immunoprecipitation

Cells were lysed in the tris-buffered saline/NP-40 (TBSN) buffer [20-mM Tris-Cl (pH 8.0), 150-mM NaCl, 0.5% NP-40, 5-mM EGTA, 1.5-mM EDTA, 0.5-mM Na_3_VO_4_, 20-mM β-glycerol phosphate, 10% glycerol and protease inhibitor cocktail]. The cell lysates were clarified by centrifugation at 15,000*g* for 20 min at 4 °C. Cleared lysates (1 mg) were added to Flag M2 agarose followed by incubation in the TBSN buffer for 1 h at 4 °C. After incubation, resins were thoroughly washed with the binding buffer and proteins bound to resin eluted in the SDS-PAGE sample buffer. A fraction of eluted sample was also analyzed by SDS-PAGE.

### Gene silencing by siRNA

A549 or 293FT/Flag-KRas^V12^ cells were transfected with siRNAs targeting Radil or with KRas siRNA using DharmaFECT I. The Radil, KRas, and nontargeting control siRNAs were purchased from Dharmacon. ON-TARGETplus Human RADIL smart pool (L-017110-00-0005), Human KRas smart pool (L-005069-00-0005), and nontargeting control pool (D-001810-10-05) were purchased from Dharmacon. To further eliminate any off-target effects, we designed additional pairs of Radil and KRas siRNAs in which control siRNAs were obtained by mutating key residues of either Radil or KRas siRNAs. Please refer to detailed descriptions in Supplemental Figure legends. Briefly, cells seeded at 50% confluence in an antibiotic-free culture medium were transfected with siRNA duplexes at a final concentration of 100 nM for 24 h. The transfection was performed at 37 °C in a humidified incubator with 5% CO_2_. Cell morphology and transfection efficiency were evaluated at 6 h after transfection. Transfections were performed in triplicate, and the experiment was repeated ≥3 times.

### Transient plasmid transfections

Twenty-four hours before the transfection, 1× 10^6^ cells were seeded in 10-cm dishes. Transfections using Lipofectamine 3000 were performed according to the manufacturer’s instructions. Transfection efficiency was estimated to be between 80 and 100% in all cases through the use of a GFP-expressing plasmid (data not shown).

### Protein extraction and immunoblotting

Total cell lysates were prepared in the TBSN buffer supplemented with a mixture of protease and phosphatase inhibitors. Protein concentrations were measured using the bicinchoninic acid protein assay reagent kit (Pierce Chemical Co). Equal amounts of protein lysates from various samples were used for SDS–PAGE analysis followed by immunoblotting. Specific signals on immunoblots were visualized using enhanced chemiluminescence (SuperSignal, Pierce Chemical Co).

Bands on immunoblots were quantified with ImageJ, a Java-based image analysis software widely used for measurement of density profiles, peak heights, as well as the peak intensity (INT) (average OD of the band, INT) or volume (average OD of the band times its area, INT∗mm2) of the band of the expected molecular weight. Specific signals shown in Figures 4, *A*, *C*, and *D*, 5, and 6*B* were quantified and plotted as histograms (please refer to Fig. S3 for details).

### Mass spectrometry

HEK293T cells were plated onto 10-cm plates at 1 × 10^6^ cells/ml. After 24 h, either pcDNA5/FRT/TO/Flag-HRas^V12^ or empty vector plasmids were transfected with Lipofectamine 3000. After a further 24 h, cells were harvested and the tagged proteins purified with 80-μl anti-M2 affinity gel according to manufacturer's instructions.

The affinity-purified proteins were loaded onto an SDS–PAGE gel to remove LC-MS–incompatible reagents. The gel plugs were excised, destained, reduced, alkylated, and subjected to proteolytic digestion with trypsin. The resulting peptides were extracted and desalted as described previously (Peled *et al*., 2017), and an aliquot of the peptides was analyzed with LC-MS coupled to a Thermo Fisher Scientific Q Exactive Mass Spectrometer operated in a data-dependent mode as described previously (34). The data were searched against a UniProt human database, using Proteome Discoverer 1.4. Please refer to Supplemental information for more details for mass spectrometry and data analyses. Please refer to Supplemental experimental procedures for additional details.

### Active Ras pull-down assay

Isolation of active Ras-GTP was performed using the Active Ras Pull-Down and Detection Kit (Thermo Fisher Scientific) following the manufacturer’s protocol. Ras abundance was measured by Western blot. Western blot analysis of RBD pull-down lysates was performed with Rabbit anti-pan RAS or Flag antibody.

### Phospho-kinase array

For phospho-kinase array analysis, cellular extracts (500 mg) were incubated with the Phospho-Kinase Array Kit (Proteome Profiler; R&D Systems, Abingdon, United Kingdom) following the manufacturer’s instructions. Densitometry values were estimated by the ImageJ software and were expressed as arbitrary units. Average signal of the pair of duplicate spots, representing each phosphorylated kinase protein, was calculated after subtraction of background values (pixel density) from negative control spots and normalization to average values from positive control spots.

### Cell invasion assays

Transwell plates (24-well insert, 8-μm pore size; Corning Costar; Corning Incorporated) were used to examine the ability of cells to invade through a Matrigel-coated filter following the protocol provided by the manufacturer. Briefly, A549 cells were transiently transfected with siRadil and or siKRas. Twenty-four hours after transfection, cells (4 × 10^4^ cells/well) were seeded onto transwell inserts and incubated at 37 °C for 12 h. Nonmigrated cells were removed from the upper face of the transwell insert using a cotton swab. Membranes of transwell insert were fixed with methanol and stained with 0.1% crystal violet. The membrane was visualized with an inverted microscope (at ×10 magnification; Nikon Corporation).

### Cell proliferation assay

Cellular proliferation was analyzed using cell counting kit-8 (Dojindo). Cells were seeded at a density of 2 × 10^3^ per well in a 96-well culture plate. Cell proliferation was measured at 1, 2, and 3 days, respectively. Serum-free culture medium (100 μl) and cell counting kit-8 solution (10 μl) were added to each well, followed by incubation at 37 °C for 1 h. The absorbance at 450 nm was measured on an SpectraMax M2 microplate reader (Molecular Devices).

### Fluorescent microscopy

Cells were washed three times with 1× PBS and then fixed with 4% paraformaldehyde for 12 min. After being penetrated by Triton X-100 (0.25%–0.5%) for 5 min, the cells were blocked by bovine serum albumin (5%) for 30 min. Cells were incubated with antibodies, for example, TRITC-conjugated phalloidin (Millipore) and mouse monoclonal vinculin (Millipore) antibodies for 45 min. Then, cells were rinsed and stained with an Alexa Fluor 488–conjugated secondary antibody (Thermo Fisher Scientific) and 2-(4-amidinophenyl)-6-indolecarbamidine dihydrochloride (Sigma-Aldrich) for 30 min.

Confocal imaging was carried out with a Leica TSC SP5 fitted with a HCX Pl APO 63 × 1.32 NA oil objective (Leica Mannheim). Images shown in figures were typically processed using levels or contrast tools using Adobe Photoshop (Adobe SI), enhancing image contrast of the different fluorescence signals in entire images.

### Statistical analysis

Each experiment was performed at least three times, and the experimental repeats were biological. The data were plotted as the mean ± SD. Student’s *t*-test was used for all comparisons. A *p* value of less than 0.05 was considered statistically significant.

## Data availability

Data described in the manuscript are intended to share with the scientific community, as well as the general public. Raw mass spectrometry data have been just deposited in the MassIVE database with the accession number MSV000085946.

## Conflict of interest

The authors declare that they have no conflicts of interest with the contents of this article.

## Supporting information

Supplemental Data

Supplemental Procedures
