# Supplementary material for: Identification of Radil as a Ras binding partner and putative activator
Source: J Biol Chem. 2021 Jan 20;296:100314. doi: 10.1016/j.jbc.2021.100314 (PMC7949112; doi:10.1016/j.jbc.2021.100314)
Supplement: Supplemental Procedures [file mmc2.pdf]

Mass Spectrometry and Data Analyses

Preparation of Samples for Mass Spectrometry

The gel bands were excised, cut into $1mm^3$ pieces and destained for 15 minutes in a 1:1 (v/v) solution of methanol and 100mM ammonium bicarbonate. The buffer was exchanged and the samples were destained for another 15 minutes. This was repeated for another 3 cycles. The gel plugs were dehydrated by washing with acetonitrile, and further dried by placing them in a SpeedVac for 20 minutes. The plugs were reduced with 100 µl of 20mM dithiothreitol (Sigma) for one hour at 57 ^{0}C at pH 7.5. The plugs were alkylated with 100 µl of iodoacetic acid (Sigma) for 45 minutes at room temperature in the dark. The gel plugs were again dehydrated by washing with acetonitrile, and further dried by placing them in a SpeedVac for 20 minutes. 250ng of sequencing grade modified trypsin (Promega) was added directly to the dried gel pieces followed by enough 100mM ammonium bicarbonate to cover the gel pieces. The gel plugs were allowed to shake at room temperature and digestion proceeded overnight. The digestion was halted by adding a slurry of R2 50 µm Poros beads (Applied Biosystems) in 5% formic acid and 0.2% trifluoroacetic acid (TFA) to each sample at a volume equal to that of the ammonium bicarbonate added for digestion. The samples were allowed to shake at 4^{0}C for 120 mins. The beads were loaded onto C18 ziptips (Millipore), equilibrated with 0.1% TFA, using a microcentrifuge for 30 s at 6,000 rpm. The beads were washed with 0.5% acetic acid. Peptides were eluted with 40% acetonitrile in 0.5% acetic acid followed by 80% acetonitrile in 0.5% acetic acid. The organic solvent was removed using a SpeedVac concentrator and the sample reconstituted in 0.5% acetic acid.

Mass Spectrometry Analysis

An aliquot of each sample was loaded onto an Acclaim PepMap trap column (2 cm x 75 µm) in line with an EASY-Spray analytical column (50 cm x 75 µm ID PepMap C18, 2 µm bead size) using the auto sampler of an EASY-nLC 1000 HPLC (Thermo Fisher Scientific) with solvent A consisting of 2% acetonitrile in 0.5% acetic acid and solvent B consisting of 80% acetonitrile in 0.5% acetic acid. The peptides were gradient eluted into a Q Exactive Mass Spectrometer (Thermo Fisher Scientific) using the following gradient: 5 - 35% in 60 min, 35 - 45% in 10 min, followed by 45 - 100% in 10 min. The gradient was held at 100% for another 10 minutes. MS1 spectra were recorded with a resolution of 70,000, an AGC target of 1e6, with a maximum ion time of 120ms, and a scan range from 400 to 1500m/z. The MS/MS spectra were collected with a resolution of

17,500, an AGC target of 5e4, maximum ion time of 120ms, one microscan, 2m/z isolation window, a Normalized Collision Energy (NCE) of 27, and included charge states from +2 to +5.

Data Processing

All acquired MS2 spectra were searched against a UniProt human database using Sequest within Proteome Discoverer 1.4 (Thermo Fisher Scientific). The search parameters were as follows: precursor mass tolerance ±10 ppm, fragment mass tolerance ±0.02 Da, digestion parameters trypsin allowing two missed cleavages, fixed modification of carboxymethyl on cysteine, variable modification of oxidation on methionine, and variable modification of deamidation on glutamine and asparagine and a 1% peptide and protein FDR cut off searched against a decoy database. The results were filtered to only include proteins identified by at least two unique peptides.

Reference:

Peled, M., Tocheva, A.S., Sandigursky, S., Nayak, S., Philips, E.A., Nichols, K.E., Strazza, M., Azoulay-Alfaguter, I., Askenazi, M., Neel, B.G., Pelzek, A.J., Ueberheide, B., Mor, A., 2017. Affinity purification mass spectrometry analysis of PD-1 uncovers SAP as a new checkpoint inhibitor. Proc. Natl. Acad. Sci. U. S. A. https://doi.org/10.1073/pnas.1710437115
