# Supplementary material for: Identification of Radil as a Ras binding partner and putative activator
Source: J Biol Chem. 2021 Jan 20;296:100314. doi: 10.1016/j.jbc.2021.100314 (PMC7949112; doi:10.1016/j.jbc.2021.100314)
Supplement: Supplemental Figures S1–S3 [file mmc3.pdf]

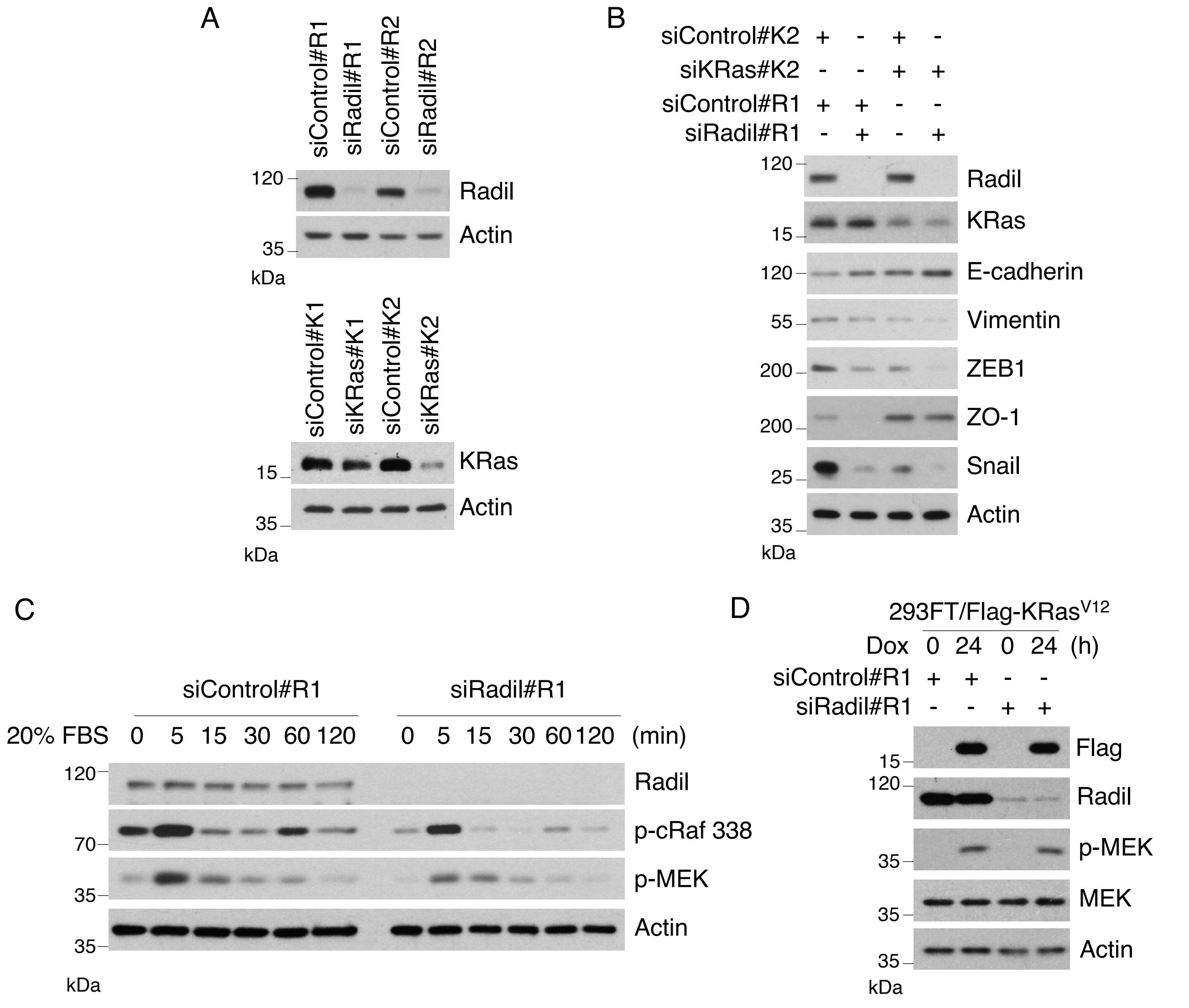
A
siControl#R1
siRadil#R1
siControl#R2
siRadil#R2
120
Radil
Actin
35
kDa
siControl#K1
siKRas#K1
siControl#K2
siKRas#K2
KRas
15
Actin
35
kDa
B
siControl#K2 + - + -
siKRas#K2 - - + +
siControl#R1 + + - -
siRadil#R1 - + - +
120
Radil
KRas
15
120
E-cadherin
55
Vimentin
200
ZEB1
200
ZO-1
25
Snail
Actin
35
kDa
C
siControl#R1
siRadil#R1
20% FBS 0 5 15 30 60 120 0 5 15 30 60 120 (min)
120
Radil
p-cRaf 338
70
p-MEK
35
Actin
35
kDa
D
293FT/Flag-KRas^{V12}
Dox 0 24 0 24 (h)
siControl#R1 + + - -
siRadil#R1 - - + +
Flag
15
120
Radil
p-MEK
35
MEK
35
Actin
35
kDa

Figure S1

A

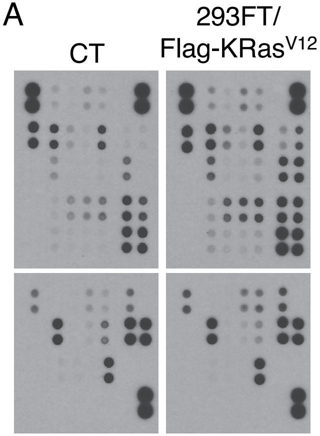

B

Position	Target/Control	Phosphorylation Site
A1, A2	Reference Spot	positive
A3, A4	p38α	T180/Y182
A5, A6	ERK1/2	T202/Y204, T185/ Y187
A7, A8	JNK 1/2/3	T183/Y185, T221/ Y223
A9, A10	GSK-3α/β	S21/S9
B3, B4	EGF R	Y1086
B5, B6	MSK1/2	S376/S360
B7, B8	AMPKα1	T183
B9, B10	Akt	S473
C1, C2	TOR	S2448
C3, C4	CREB	S133
C5, C6	HSP27	S78/S82
C7, C8	AMPKα2	T172
C9, C10	β-Catenin	total
D1, D2	Src	Y419
D3, D4	Lyn	Y397
D5, D6	Lck	Y394
D7, D8	STAT2	Y689
D9, D10	STAT5a	Y694
E1, E2	Fyn	Y420
E3, E4	Yes	Y426
E5, E6	Fgr	Y412
E7, E8	STAT6	Y641
E9, E10	STAT5b	Y699
F1, F2	Hck	Y411
F3, F4	Chk-2	T68
F5, F6	FAK	Y397
F7, F8	PDGF Rβ	Y751
F9, F10	STAT5a/b	Y694/Y699
G1, G2	Reference Spot	positive
G3, G4	PRAS40	T246
G9, G10	PBS	negative

C

Position	Target/Control	Phosphorylation Site
A17, A18	Reference Spot	positive
A13, A14	p53	S392
B11, B12	Akt	T308
B13, B14	p53	S46
C11, C12	p70 S6 Kinase	T389
C13, C14	p53	S15
C15, C16	c-Jun	S63
D11, D12	p70 S6 Kinase	T421/S424
D13, D14	RSK1/2/3	S380/S386/S377
D15, D16	eNOS	S1177
E11, E12	STAT3	Y705
E13, E14	p27	T198
E15, E16	PLC-γ1	Y783
F11, F12	STAT3	S727
F13, F14	WNK1	T60
F15, F16	PYK2	Y402
G11, G12	HSP60	total
G17, G18	PBS	negative

D

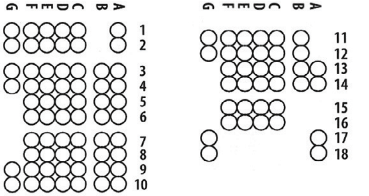

E

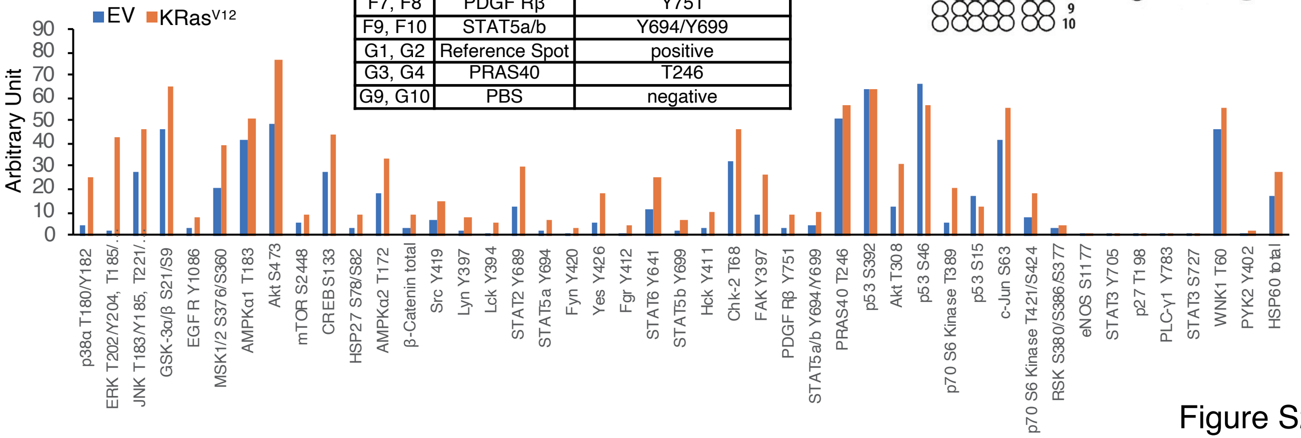

Figure S2

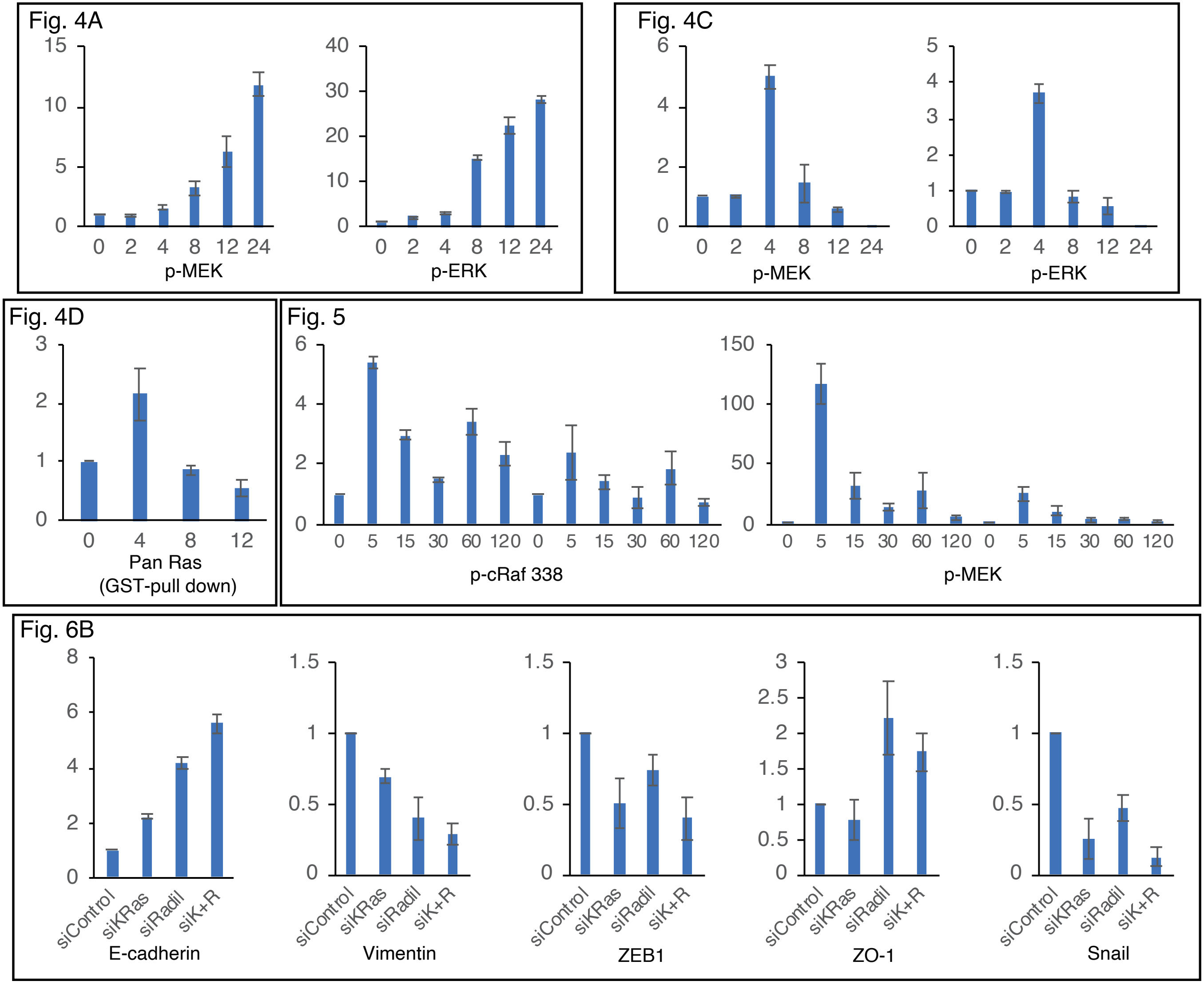

Fig. 4A
p-MEK
p-ERK
0 2 4 8 12 24
Fig. 4C
p-MEK
p-ERK
Fig. 4D
Pan Ras
(GST-pull down)
0 4 8 12
Fig. 5
p-cRaf 338
p-MEK
0 5 15 30 60 120 0 5 15 30 60 120
Fig. 6B
siControl
siKRas
siRadil
siK+R
E-cadherin
Vimentin
ZEB1
ZO-1
Snail

Figure S3

Figure Legends (Supplemental Figures)

Figure S1. Specific down-regulation of Radil or KRas via RNAi. (A) Cells transfected siRNAs targeting two different stretches of Radil (upper panel) or KRas (lower panel). Control siRNAs were made by mutating key residues within targeting siRNAs. Sequences corresponding to these siRNAs are as follows: siRadil#R1 5' CCAAAGAACUAGCAGAGAAUU 3'; siRadil#R2 5' GGAGAAAACCAAAGAACUAUU 3'; siControl#R1 5' CCAAAGAUCUAACAGAGAAUU 3'; siControl#R2 5' GGUGAAUACCAAAGAACUAUU 3'; siKRas#K1 5' CGAAUAUGAUCCAACAAUAUU 3'; siKRas#K2 5' AGCAAGAAGUUAUGGAAUUUU 3'; siControl#K1 5' CGAAUUUGAUCGAACAAUAUU 3'; siControl#K2 5' AGCAAGUAGUAAUGGAAUUUU 3'. (B) A549 cells transfected with Radil siRNAs (siRadil#R1) and/or KRas siRNAs (siRNA#K2) for 24 h. Respective control siRNAs (siControl#K2 or siControl#R1) as described in A were also used for transfection. Equal amounts of cell lysates were then blotted for Radil, KRas, E-cadherin, Vimentin, ZEB1, ZO-1, Snail, and actin. (C) A549 cells were transfected with Radil siRNAs (siRadil#R1) or control siRNAs (siControl#R1) for 24 h, starved in low (0.2%) FBS medium for 16 h, and then fed 20% serum for various times as indicated. Equal amounts of cell lysates were then blotted for Radil, phospho-cRaf 338, phospho-MEK, and actin. (D) 293FT cells engineered to express inducible Flag-Radil (293FT/Flag-Radil) were transfected with Radil siRNA (siRNA#R1) or control siRNA (siControl#R1) for 24 h and then treated with Dox for 24 h. Equal amounts of cell lysates were blotted for Flag, Radil, phospho-MEK, total MEK, and β-actin.

Figure S2. Phospho-kinase profiling via dot blotting analyses. (A) 293FT cells engineered to express inducible Flag tagged $KRas^{V12}$ (293FT/Flag-$KRas^{V12}$) and 293FT control (CT) cells were starved in low FBS (0.2%) medium for 16 h, and then treated with doxycycline (Dox) for 24 h. Equal amounts of cell lysates were subjected to phospho-kinase profiling using the dot blot approach. Paired dot blot results are shown. (B-D) Detailed descriptions of protein kinases and their sites of phosphorylation being analyzed, as well as locations/orientations of each kinase on the blots. (E) Quantifications of kinase signals on the dot blots.

Figure S3. Specific signals presented in Figures 4, 5 and 6 were quantified. Results were plotted in histograms for comparison.
